# Fibromyalgia is associated to receiving chronic medications beyond appropriateness: a cross-sectional study

**DOI:** 10.1007/s00296-016-3568-2

**Published:** 2016-09-23

**Authors:** Javier Rivera, Miguel A. Vallejo

**Affiliations:** 1 Rheumatology Unit-IPR, Hospital Universitario Gregorio Marañón, c/Francisco Silvela 40, 28028 Madrid, Spain; 2 Department of Clinical Psychology, National Distance Education University (UNED), c/Juan del Rosal 10, 28040 Madrid, Spain

**Keywords:** Fibromyalgia, Comorbidity, Drug treatment, Prescription appropriateness

## Abstract

The objectives of this study are to describe appropriateness and drug treatment of comorbidities in fibromyalgia (FM). Cross-sectional study of a group of patients. Number of drugs, indication, duration and appropriateness of prescriptions were evaluated. Patients were classified as: group 1, (FM/FM) previous FM diagnosis and fulfilling criteria; group 2, (noFM/noFM) other diagnosis and not fulfilling criteria; and group 3, (noFM/FM) other diagnosis but fulfilling criteria. Drugs were classified into drugs for nervous system, analgesics/NSAID and drugs for other comorbidities. Appropriateness was evaluated following clinical therapeutic guidelines. A total of 159 patients were included in the study and classified into group 1, with 59 patients; group 2, with 67 patients; and group 3, with 33 patients. Group 1 received a greater number of different drugs and for a longer period of time, there were less severe comorbidities and more unjustified treatments. No difference was found between the other two groups. Major opioids were only consumed in group 1. Also, in group 1, 45.8 % of patients were attended in psychiatry versus 15.6 % in group 3 and 3 % in group 2. The number of somatic symptoms correlated significantly with the number of drugs. Nervous system treatments were of shorter duration than other drug treatments. There was no difference in severe comorbidities. Comorbidities in FM are similar to those of other patients, but they receive more drugs and for a longer period of time. Drugs for nervous system comorbidities are introduced later, when other somatic symptoms are already treated. In patients with FM the treatments for mild comorbidities are not well justified.

## Introduction

It is widely known that fibromyalgia (FM) is a condition with numerous symptoms that virtually involve any organ or system [1]. The term comorbidity is used when “more than one disease or condition is present in the same person at the same time” [2]. Therefore, all those clinical conditions associated with FM may be considered as comorbidities. Some studies have shown that FM is one of the rheumatic diseases with a greater number of comorbidities—more than systemic lupus erythematosus or rheumatoid arthritis [3, 4]—and that it produces a considerable impairment in the quality of life [5].

In addition, it has been shown that the association of multiple chronic clinical conditions in the same patient is very frequent and that the consequences are more important than the simple addition of separate conditions [6]. Managing this situation effectively requires a multidisciplinary approach for treating all the problems together rather than separately. A recent study [7] describes the problem of multiple clinical conditions in FM with seven or more chronic conditions in each patient. The study also highlights the risk of polypharmacy and the possibility of an impairment of clinical symptoms due to drug interactions.

Most symptoms present in patients with FM are very common and may be seen with or without the diagnosis of FM [8]. However, it is unknown whether FM diagnosis determines the treatment of these symptoms [9].

To explain this polymedication in patients with FM, we have hypothesized if the types of comorbidities, the drugs used for treating them and the appropriateness of these drugs are different in FM in comparison with other patients.

The analysis of the drugs used for treating FM patients may be considered as an estimation of the number of associated comorbidities. It also allows to assess the importance of the comorbidity as well as the impact on the disease. In addition, assessing the appropriateness of the prescribed drugs may be very useful to reduce the number of drugs and the risk of interactions and adverse events.

The aims of this study are the following: first, to describe the drugs prescribed for treating all the comorbidities present in FM patients and, second, to evaluate the appropriateness of these treatments.

## Methods

### Design

Cross-sectional study.

### Patients

The study was performed in a Rheumatology Clinic in the Comunidad Autónoma of Madrid (Spain). This clinic attends all types of rheumatic diseases and is also a reference centre for FM patients.

Patients consecutively referred from general practitioners for the first time were considered for the study. Patients with malignancies, infectious or inflammatory diseases that might affect clinical manifestations were discharged. Only 18–65-year-old native Spanish women who had given their informed consent were included in the study.

### Drugs prescription and data collection

Clinical records—both of primary care and specialized medicine—were retrieved and registered through HORUS (a sanitary management program of the Comunidad Autónoma of Madrid), as well as the number of drugs prescribed and the duration of the treatment at the moment of their first visit. Patients were divided into three groups according to whether they had a diagnosis of FM or fulfilled ACR classification criteria [10] for FM as follows:

- Group 1 (FM/FM). Patients referred with a previous diagnosis of FM and also fulfilling ACR criteria.
- Group 2 (noFM/noFM). Patients referred with other diagnosis different from FM and not fulfilling ACR criteria.
- Group 3 (noFM/FM). Patients referred with other diagnosis different from FM but fulfilling ACR criteria.

Only drugs prescribed by a physician were considered for the study. Herbal drugs and other pharmaceuticals remedies were not included. The duration in months from the beginning of the treatment was quantified with the exception of NSAID and common analgesics because they are prescribed by multiple reasons and used on demand.

Different drugs for treating all comorbidities were classified within three categories:

(A) Drugs for nervous system (NS) comorbidities. Prescribed for any NS symptoms, sleep disorders or psychiatric diseases. These include antidepressants, anticonvulsants, benzodiazepines, hypnotics, antipsychotics and dopaminergic agonists.
(B) Analgesics and nonsteroidal anti-inflammatory drugs (NSAID). Analgesics were divided into common analgesics such as paracetamol, metamizole and ibuprofen; minor opioids such as tramadol and codeine, and major opioids that include fentanyl, tapentadol, oxycodone, buprenorphine and morphine. Although metamizole and ibuprofen are NSAID, they are usually indicated for analgesia.
(C) Drugs and supplements for treating other comorbidities. Herein were included the remaining drugs. Although continuous positive airway pressure (CPAP) is not a drug, it is the main therapeutic measure for treating sleep apnoea and was included in this category.

To evaluate the appropriateness of a drug treatment, clinical therapeutical guidelines frequently used in Spain were employed. Thus, the following drug treatments were assessed: lipid alterations [11], high blood pressure [12], osteoporosis [13], diabetes mellitus [14] and hypothyroidism [15]. To evaluate the appropriateness of a gluten-free diet, an intestinal biopsy with presence of MARSH 3 alterations was required [16]. For a lactose intolerance, the presence of alterations in any of the tests used to diagnose this entity (hydrogen test, faecal acidosis or intestinal biopsy) was required. For using CPAP, the presence of nocturnal apnoea demonstrated in a sleep laboratory analysis was required. The necessity of coenzyme Q10 supplements was established if muscular biopsy was compatible with this deficit.

In vitamin D, B12, other vitamins, iron and folic acid deficiencies, low serum levels were required to evaluate the appropriateness of supplementation.

In those treatments mostly depending on the facultative criteria to initiate the drug treatment, the appropriateness of the drug was not evaluated. These included drugs for treating FM, any type of NS comorbidities, gastric protectors, analgesics and NSAID.

Among those comorbidities in which it was possible to evaluate the appropriateness of the treatment, comorbidities were classified as severe or mild following the researchers’ criteria. Hypercholesterolaemia, high blood pressure, osteoporosis, diabetes mellitus, hypothyroidism and sleep apnoea were considered as severe due to frequent and important complications such as cardiovascular events, fracture, metabolic decompensations or sudden death. However, vitamin D, other vitamins, iron, folic acid or Q10 deficiencies, coeliac disease and lactose intolerance were considered as mild comorbidities because complications are seen more rarely.

### Statistical analysis

Nominal values were described by means of a frequency analysis, and continuous variables were described with the mean and the standard deviation. Level of significance was considered at *p* < .05.

To compare differences between groups, a Chi-square test was used for nominal variables and analysis of the variance for continuous variables. Bonferroni correction was used for the post hoc analysis for comparing differences between two groups.

Pearson correlation coefficient was used for analysis of correlation between continuous variables and rho Spearman correlation coefficient for non-parametric correlations.

## Results

Between May and December 2015, a total of 353 patients were consecutively referred at the Rheumatology Clinic. One-hundred and ninety-four were excluded from the study for the following reasons: older than 65 years, 100; males, 53; foreigners, 26; inflammatory diseases, 5; malignancies, 5, and infectious diseases, 5 patients.

A total of 159 patients satisfied inclusion criteria for the study. Sixty patients were previously diagnosed of FM and were referred for treatment and control of the disease, and 99 patients were referred for study, none of which had been previously diagnosed of FM.

After applying ACR 2010 classification criteria, patients were split into three groups, as shown on Table 1. One patient with a previous diagnosis of FM did not fulfil ACR criteria at the first visit and was not included in the analysis.

**Table 1:** Groups of patients after applying ACR 2010 classification criteria [10]. Variables conforming criteria, age and the total number of drugs consumed by the patients on first visit

	Group 1 FM/FM (mean ± SD)	Group 2 noFM/noFM (mean ± SD)	Group 3 noFM/FM (mean ± SD)	Difference between groups *p*
Number of patients	59	67	32	–
Pain index (0–19)^(¶)^	14.54 ± 3.43^(a)^	4.63 ± 2.54^(b)^	10.19 ± 3.26^(c)^	<.001
Symptoms severity (0–12)^(¶)^	7.32 ± 1.47^(a)^	3.06 ± 1.64^(b)^	6.03 ± 1.63^(b)^	<.001
Somatic symptoms (0–41)^(¶)^	20.46 ± 5.98^(a)^	4.61 ± 1.99^(b)^	13.50 ± 4.71^(c)^	<.001
Age (yrs.)	48.07 ± 9.29^(a)^	52.36 ± 9.64	49.44 ± 8.79	<.035
All drugs consumed total number	6.81 ± 3.54^(a)^ 402	2.3 ± 2.23^(b)^ 154	3.69 ± 2.74 118	<.001

Group 1 (FM/FM): diagnosis of FM and fulfilling ACR criteria; group 2 (noFM/noFM): other diagnosis and not fulfilling ACR criteria; group 3 (noFM/FM): other diagnosis and fulfilling ACR criteria

(^¶^) Variables conforming ACR 2010 criteria and score range

*Post hoc* analysis: ^(a)^ significant difference between groups 1 and 2; ^(b)^ significant difference between groups 2 and 3; ^(c)^ significant difference between groups 1 and 3. Significant values at *p* < .05

Final diagnoses in the patients of group 2 were as follow: osteoarthritis at different locations, 34; osteoporosis, 20; acute lumbalgia, 4; tendonitis, 2; primary Raynaud, 2; muscular spasms, 2; skin psoriasis, 1; metatarsal fracture, 1; and discal herniation, 1. Variables conforming ACR criteria were different between groups with greater scores in group 1 followed by group 3 and group 2 (Table 1).

There was a significant difference between groups in the total number of drugs consumed.

A significant positive correlation was found between the number of somatic symptoms and the number of drugs consumed (*r* = 0.622, *p* < .001). A weakly but significant correlation was also found between the age and the number of drugs consumed (*r* = 0.187, *p* = .018).

With respect to drugs used for treating NS comorbidity, there was a significant difference between groups in the total number of drugs consumed, as well as for all types of NS drugs, with the exception of antipsychotics (Table 2).

**Table 2:** Drugs for treating NS comorbidities

	Group 1 FM/FM Number of patients (%)	Group 2 noFM/noFM Number of patients (%)	Group 3 noFM/FM Number of patients (%)	Difference between groups *p*
Benzodiazepines and hypnotics	43 (72.9)^(a)^	14 (20.9)^(b)^	13 (40.6)^(b)^	<.001
Antidepressants	38 (62.7)^(a)^	8 (11.9)	5 (15.6)^(b)^	<.001
Anticonvulsants	19 (32.2)^(a)^	1 (1.5)^(b)^	5 (15.6)	<.001
Antipsychotics	3 (5.1)	1 (1.5)	1 (3.1)	ns
Duration in months (mean ± SD)	94.22 ± 100.01^(a)^	25.19 ± 72.98	40.40 ± 77.89^(c)^	<.001
NS drugs consumed (mean ± SD) Total number	2.25 ± 1.63^(a)^ 133	0.37 ± 0.81 25	0.88 ± 1.12^(c)^ 28	<.001

Group 1 (FM/FM): diagnosis of FM and fulfilling ACR criteria; group 2 (noFM/noFM): other diagnosis and not fulfilling ACR criteria; group 3 (noFM/FM): other diagnosis and fulfilling ACR criteria

*ns* non-significant

*Post hoc* analysis: ^(a)^ significant difference between groups 1 and 2; ^(b)^ significant difference between groups 2 and 3; ^(c)^ significant difference between groups 1 and 3. Significant values at *p* < .05

In group 1, 86.4 % of the patients received any type of NS drugs versus 28.4 % in group 2 and 46.9 % in group 3 (*p* < .001). Moreover, 45.8 % of the patients in group 1 were also attended in psychiatry versus 3 % in group 2 and 15.6 % in group 3 (*p* < .001).

With respect to analgesics and NSAID, there were also differences between groups for all these drugs (Table 3). Major opioids were only consumed for patients in group 1 (Table 3).

**Table 3:** Analgesics and NSAID

	Group 1 FM/FM Number of patients (%)	Group 2 noFM/noFM Number of patients (%)	Group 3 noFM/FM Number of patients (%)	Difference between groups *p*
NSAID	40 (67.8)^(a)^	13 (19.4)	9 (28.1)^(c)^	<.001
Common analgesics	35 (59.3)^(a)^	20 (29.9)	16 (50.0)	<.003
Tramadol + paracetamol	8 (13.6)^(a)^	2 (3.0)	1 (3.1)	<.042
Tramadol	6 (10.2)^(a)^	0 (0.0)^(b)^	4 (12.5)	<.018
Major opioids	8 (13.6)^(a)^	0 (0.0)	0 (0.0)^(c)^	<.001
Duration in months^(¶)^ (mean ± SD)	5.28 ± 16.03^(a)^	0.0 ± 0.0	1.40 ± 5.42	<.05
Analgesics and NSAID consumed (mean ± SD) total number	1.85 ± 0.94^(a)^ 109	0.58 ± 0.76^(b)^ 39	1.06 ± 1.04^(c)^ 34	<.001

Group 1 (FM/FM): diagnosis of FM and fulfilling ACR criteria; group 2 (noFM/noFM): other diagnosis and not fulfilling ACR criteria; group 3 (noFM/FM): other diagnosis and fulfilling ACR criteria

(^¶^) Duration for tramadol and major opioids

*Post hoc* analysis: ^(a)^ significant difference between groups 1 and 2; ^(b)^ significant difference between groups 2 and 3; ^(c)^ significant difference between groups 1 and 3. Significant values at *p* < .05

With respect to drugs used for treating other comorbidities, group 1 received more drugs and there was no difference between groups 2 and 3 (Table 4). Gastric protectors, vitamin D supplements, antimigraine agents, lactose-free diet and betahistine for dizziness were more common in group 1, while biphosphonates were more common in group 2.

**Table 4:** Drugs, supplements and other measures for treating other comorbidities

	Group 1 FM/FM Number of patients (%)	Group 2 noFM/noFM Number of patients (%)	Group 3 noFM/FM Number of patients (%)	Difference between groups *p*
Gastric protectors	36 (61.0)^(a)^	12 (17.9)^(b)^	15 (46.9)	<.001
Thyroid hormone	9 (15.3)	13 (19.4)	6 (18.8)	ns
Antihypertensive	14 (23.7)	10 (14.9)	4 (14.5)	ns
Vitamin D supp.	15 (25.4)^(a)^	5 (7.5)	3 (9.4)	<.011
Statins	6 (10.2)	11 (16.4)	6 (18.8)	ns
Calcium + Vit.D supp.	6 (10.2)	12 (17.9)	4 (12.5)	ns
Bronchodilators	6 (10.2)	2 (3.0)	3 (9.4)	ns
Antimigraine agents	7 (11.9)^(a)^	1 (1.5)	1 (3.1)	<.034
Antidiabetics	4 (6.8)	4 (6.0)	1 (3.1)	ns
Bisphosphonates	1 (1.7)^(a)^	7 (10.5)	0 (0.0)	<.028
Polivitamins supp.	5 (8.5)	1 (1.5)	2 (6.3)	ns
Antihistamines	3 (5.1)	2 (3.0)	3 (9.4)	ns
Iron supp.	3 (5.1)	1 (1.5)	2 (6.3)	ns
SYSADOA	5 (8.5)	0 (0,0)	1 (3.1)	<.045
Domperidone	4 (6.8)	0 (0,0)	1 (3.1)	ns
Gluten-free diet	4 (6.8)	1 (1.5)	0 (0.0)	ns
Lactose-free diet	4 (6.8)^(a)^	0 (0.0)	0 (0.0)	<.032
Betahistine	4 (6.8)^(a)^	0 (0.0)	0 (0.0)	<.032
Various^(¶)^	24	8	4	–
Duration in months (mean ± SD)	154.03 ± 180.10^(a)^	71.46 ± 105.67	112.78 ± 206.11	<.016
Other comorbidities drugs consumed (mean ± SD) Total number	2.71 ± 2.19^(a)^ 160	1.34 ± 1.50 90	1.75 ± 1.68 56	<.001

Group 1 (FM/FM): diagnosis of FM and fulfilling ACR criteria; group 2 (noFM/noFM): other diagnosis and not fulfilling ACR criteria; group 3 (noFM/FM): other diagnosis and fulfilling ACR criteria

*Post hoc* analysis: ^(a)^ significant difference between groups 1 and 2; ^(b)^ significant difference between groups 2 and 3; ^(c)^ significant difference between groups 1 and 3. Significant values at *p* < .05

*ns* non-significant, *Supp*. supplements, *SYSADOA* symptomatic slow action drugs for osteoarthritis

(^¶^) Between parents, means number of patients treated. Folic acid supp. (4), metoclopramide (3), beta blockers (3), nasal decongestants (3), continuous positive airway pressure (CPAP) (2), antispasmodics (2), diuretics (2), acetylsalicylic acid (2), hormone replacement therapy (2), fructose-free diet (1), diosmin (1), carbamazepine (1), tamoxifene (1), Q10 supp. (1), rotigotine (1), clebopride (1), hydroaltesone (1), tranexamic acid (1), ursodeoxycholic acid (1), sibutramine (1), calcitonin (1), salazopyrin (1)

In the total number of patients, there was a positive correlation between the use of gastric protectors and the number of drugs consumed for treating all comorbidities (rho Spearman = 0.648, *p* < .0001).

The duration of the treatments for all types of comorbidities was significantly longer in group 1 than in the two other groups. There were no differences between groups 2 and 3 (see Tables 2, 3, 4).

The analysis of the duration of all types of treatments in patients of group 1 showed that benzodiazepines, antidepressants and anticonvulsants had a shorter median duration than other drugs for treating other comorbidities (Table 5).

**Table 5:** Duration in months of different treatments in patients of group 1 (FM/FM). Benzodiazepines, antidepressants and anticonvulsants are in bold in the table. Only those treatments administered to three or more patients are shown in the table

	Number of patients with treatment	Duration of treatment in months (mean ± SD)
Thyroid hormone	9	152.00 ± 116.34
Lactose-free diet	4	137.00 ± 168.48
Gluten-free diet	4	114.00 ± 164.68
Polivitamins supp.	5	84.80 ± 155.00
Betahistine	4	78.25 ± 74.59
Antidiabetics	4	74.75 ± 52.72
Bronchodilators	6	71.00 ± 38.77
Gastric protectors	35	69.40 ± 65.93
**Benzodiazepines**	**43**	**66.55** **±** **73.09**
Statins	6	66.00 ± 31.06
**Antidepressants**	**37**	**53.89** **±** **53.34**
Antihypertensives	14	50.28 ± 47.51
Calcium + Vit.D supp.	6	43.50 ± 44.24
Domperidone	4	42.00 ± 28.56
Antimigraine agents	7	40.71 ± 35.80
**Anticonvulsants**	**19**	**37.00** **±** **38.55**
Folic acid supp.	3	29.00 ± 23.30
Antihistamines	3	17.16 ± 26.73
Vitamin D supp.	15	16.40 ± 20.77
SYSADOA	5	13.10 ± 20.01
Iron supp.	3	5.66 ± 5.50
Major opioid	6	4.00 ± 15.33
Tramadol	5	1.29 ± 5.69

With respect to the analysis of the severity of comorbidities, there was no significant difference between groups in the number of severe comorbidities. The comparison between the number of appropriate treatments in severe comorbidities also showed no significant differences between groups (Table 6).

**Table 6:** Types of comorbidities and appropriateness of the treatments

	Group 1 FM/FM (mean ± SD)	Group 2 noFM/noFM (mean ± SD)	Group 3 noFM/FM (mean ± SD)	Difference between groups *p*
Number of patients	59	67	32	
Severe comorbidities Total number	0.61 ± 0.89 36	0.67 ± 0.97 45	0.53 ± 0.72 17	ns
Unjustified treatments of severe comorbidities Total number	0.08 ± 0.33 5	0.16 ± 0.37 11	0.09 ± 0.29 3	ns
Mild comorbidities Total number	0.61 ± 0.94^(a)^ 36	0.13 ± 0.34 9	0.22 ± 0.55^(c)^ 7	<.0001
Unjustified treatments of mild comorbidities Total number	0.37 ± 0.69^(a)^ 22	0.04 ± 0.20 3	0.19 ± 0.47 6	<.001

Group 1 (FM/FM): diagnosis of FM and fulfilling ACR criteria; group 2 (noFM/noFM): other diagnosis and not fulfilling ACR criteria; group 3 (noFM/FM): other diagnosis and fulfilling ACR criteria

*ns* non-significant

*Post hoc* analysis: ^(a)^ significant difference between groups 1 and 2; ^(b)^ significant difference between groups 2 and 3; ^(c)^ significant difference between groups 1 and 3. Significant values at *p* < .05

However, in the case of mild comorbidities, there was a significant difference between groups with a greater number of comorbidities in patients of group 1 (Table 6). Moreover, the number of appropriate treatments was also significantly different with more unjustified treatments in group 1 versus the two other groups. There were no differences between groups 2 and 3 (Table 6).

## Discussion

The types of comorbidities found in patients with FM were similar to the ones in the control patients without FM. The most relevant differences were the higher number and the longer duration of the treatments found in patients with FM in this study.

Drugs for treating NS comorbidities, migraines, dizziness, gastric protectors and the use of analgesics were more common in patients with FM, as described in previous studies [7], but these clinical conditions are also commonly present in many patients without FM.

Drugs for treating potential severe comorbidities such as high blood pressure, hypothyroidism or high cholesterol levels were the same as in controls without FM showing that these types of comorbidities are not increased in FM patients. Similar findings were found in a recent systematic revision [17] where the presence of severe comorbidity in FM was analysed but no clear association was found.

However, drugs for treating mild comorbidities such as vitamin D or other vitamins, iron, folic, Q10 deficiencies, coeliac disease and lactose intolerance were found significantly more frequent in patients with FM. It is known that the prescription of these types of treatment depends on the facultative criteria and it is not usually based on clinical practice guidelines recommendations.

The difference between comorbidity and symptom is not well defined [18]. Most drugs consumed by patients with FM are prescribed for treating symptoms that are inherent to the principal clinical picture of FM and for this reason they are not considered as comorbidities by some authors [4]. Positive correlation found in this study between somatic symptoms and the number of drugs prescribed indicates that the more symptomatic the disease is the higher is the number of drugs prescribed.

For example, in rheumatoid arthritis the extra-articular manifestations are considered as clinical manifestations by some authors or as comorbidities by others [19]. The treatment used to be almost the same. However, considering clinical manifestations as symptoms or as comorbidities in FM—a multisymptomatic disease—can have considerable consequences. In the first case, the main treatment of FM can be sufficient to improve symptoms but, in the second case, the probability of prescribing new drugs is high.

Separate treatment of clinical manifestations does not guarantee an improvement in the disease and may increase the risk of drug interactions and adverse events adding more clinical manifestations or aggravating them [7]. Some studies have shown that some clinical manifestations such as dizziness—a common symptom in patients with FM—are positively correlated with the number of consumed drugs [20].

Positive correlation between the use of gastric protectors and the total drugs consumed by our patients probably indicate the need to add more drugs to prevent complications when many other treatments are already used. This clearly contributes to increase polymedication.

Opioids prescription in patients with FM has increased drastically over the past years. However, it is well known that opioid prescribing decision is not based only on patient–physician interaction but also on other factors that may explain the wide geographic variation found in some studies [21].

In our study, 13.6 % of patients with FM were taking major opioids and 74 % consumed benzodiazepines. Recently, it has been shown that combined consumption of these two drugs clearly increases the risk of serious interactions and can multiply by 15 the risk of death [22]. Although there is no conclusive evidence against these two drugs in the treatment of FM, recent clinical guidelines in FM advise against their use by their deleterious adverse events [23, 24].

Psychiatric symptoms are very frequent and may be seen in two-thirds of the patients with FM, mostly depressive syndromes [25]. The question of whether psychiatric symptoms precede the development of FM or appear along the course of the disease is frequently considered and does not have a good answer. But it is a very important issue when planning the treatment and follow-up of these patients.

Treatment with benzodiazepines and antidepressants has a shorter duration than with other drugs in this study, and they are prescribed when other comorbidities are already being treated. Additionally, 45 % of patients with a previous diagnosis of FM (group 1) versus 15 % of the patients with other diagnosis but fulfilling ACR criteria for FM (group 3) were also attended by psychiatrists. These data suggest that psychiatric comorbidity appears later in the course of the disease when other comorbidities and treatments have already started. It also means that in multisymptomatic patients an early psychological therapy focused on relaxation techniques, learning and acquisition of adequate coping strategies to manage initial somatic manifestations may be a great help to reduce NS drugs [26].

The appropriateness of the severe comorbidity treatments has shown that criteria for treatment were well established following clinical practice guidelines. However, for mild comorbidities, patients with FM received a greater number of treatments which were not well justified. This means that some drugs used for treating some clinical symptoms should be eliminated.

Patients’ demand for an efficient solution of their symptoms put a lot of pressure on the attending physician. In the absence of more appropriate resources—such as the psychological therapy mentioned before—the option of a drug prescription is an evident risk that should be avoided [27, 28].

Those patients who had not been previously diagnosed of FM but fulfilled ACR criteria (group 3) present some interesting features. The total number of drugs consumed, duration of treatments, presence of mild comorbidities and treatment appropriateness are the same as those of patients that do not have FM (group 2). Moreover, in this group, the analysis of the duration of the different treatments shows that benzodiazepines are the first drug prescribed for treating NS comorbidity.

This group is also interesting because it is composed of patients in the initial phases of the disease. On the one hand, they have many somatic symptoms and may be diagnosed of FM, but, on the other hand, they are not still treated with many drugs. Identifying these patients is very important in order to control the evolution of the disease and to avoid unnecessary use of drugs and their negative consequences.

In rheumatoid arthritis, some recommendations for treatment and control of the comorbidities have been recently established, due to the increased mortality risks [19]. In the case of FM, establishing treatment criteria and recommendations for somatic symptoms and other comorbidities is also important, in this case to avoid polimedication, drug interactions and symptoms exacerbation.

This study has several limitations. The cross-sectional design of the study describes the situation of the patients compared with that of controls, but it does not shed light on the origin or the consequences of the problem. The lack of a control group with individuals of the general population does not allow a better evaluation of the drugs prescribed for treating comorbidities in these patients.

The fact that this study has been performed in only one centre specialized in FM management does not allow to generalize the results to other primary care centres or other types of patients.

The strong point of the paper is that its design and results allow a clear visualization of the FM spectrum, from mild clinical pictures with the possibility of early interventions and possible prevention up to severe cases.

## Conclusions

The types of comorbidities in patients with FM are similar to the ones presented by other patients without the disease. The main differences are quantitative, patients with FM received more drugs and for a longer period of time for treating these comorbidities.

Drugs for treating NS comorbidities appear later, when other somatic symptoms are already treated. There are also mild comorbidities where the treatment with drugs is not well justified.
